# A least action principle for interceptive walking

**DOI:** 10.1038/s41598-021-81722-6

**Published:** 2021-01-26

**Authors:** Soon Ho Kim, Jong Won Kim, Hyun Chae Chung, MooYoung Choi

**Affiliations:** 1 Department of Physics and Astronomy and Center for Theoretical Physics, Seoul National University, Seoul, 08826 Korea; 2 Department of Healthcare Information Technology, Inje University, Gimhae, 50834 Korea; 3 Department of Sport and Exercise Sciences, Kunsan National University, Gunsan, 54150 Korea; 4 Present Address: Brain Science Institute, Korea Institute of Science and Technology, Seoul, 02792 Korea

**Keywords:** Applied physics, Human behaviour

## Abstract

The principle of least effort has been widely used to explain phenomena related to human behavior ranging from topics in language to those in social systems. It has precedence in the principle of least action from the Lagrangian formulation of classical mechanics. In this study, we present a model for interceptive human walking based on the least action principle. Taking inspiration from Lagrangian mechanics, a Lagrangian is defined as effort minus security, with two different specific mathematical forms. The resulting Euler–Lagrange equations are then solved to obtain the equations of motion. The model is validated using experimental data from a virtual reality crossing simulation with human participants. We thus conclude that the least action principle provides a useful tool in the study of interceptive walking.

## Introduction

Principles of least action play a fundamental role in many areas of physics. They were preceded by Fermat’s principle or the principle of least time in geometrical optics^1^. In classical mechanics, equations of motion can be generated from Maupertuis’s principle of least action or the closely related Hamilton’s principle^2,3^. Theories of modern physics, including quantum mechanics and general relativity, also have formulations in terms of principles of least action^4,5^. Such formulations mostly adopt calculus of variations in which the dynamics selected by the theory, e.g. the path taken by a particle, is governed by the stationarity of the suitably defined action. In the context of classical mechanics, the action is given by the time integral of the Lagrangian along the path of motion.

In the study of human behavior, these principles have inspired the principle of least effort, which was used to explain the power-law form of the rank-frequency distribution of words in the English language^6^, and subsequently a diverse range of phenomena such as crowd behavior^7^ and even mental effort^8^. These studies have found predictable patterns in distributions arising from human behaviors, which individually are variable and unpredictable. However, it is reasonable to expect that there should typically be a tradeoff between effort and other quantities. Therefore, a more complete description of human behaviors may plausibly be provided by a least action principle in which effort is one component. In this report, we take this approach and propose a principle of least action, which is used for modeling human walking behavior.

The study of locomotion requires an integrative approach^9^, and human bipedal locomotion in particular has a long evolutionary history which resulted in a uniquely economical cost of transport^10^. Human walking exhibits a parabolic curve when the cost of transport is plotted against speed; the optimum walking speed is related to the pendular mechanism of human walking^11,12^. Here we are concerned with trajectories of interceptive walking^13–15^. It is widely observed that during acts of interception, certain paths tend to be taken, despite the inherent variability of human action. This study presents a quantitative model that characterizes a wide range of such individual walking trajectories based on a few key assumptions, thus capturing the essential features of interceptive walking. In doing so, we ignore the specific biomechanics^12,16^ and gait patterns^17–19^ of walking, which are themselves subjects of active study.

In the following sections, we first propose the Lagrangian mechanics of interceptive walking by defining a Lagrangian and solving the resulting Euler–Lagrange equation, which yields the path of stationary action. Inspired by the principle of least effort, we postulate that the Lagrangian consists of effort as well as a quantity we call security. Two specific forms of the Lagrangian are considered and their respective equations of motion are derived. Then the equations of motion are verified by fitting the solutions to positional time series data from a virtual reality crossing experiment. The experiment simulates a road crossing situation in which a pedestrian should cross between two moving vehicles, hence intercepting the gap^20–23^. Finally, we discuss the meaning of the model and fitting parameters.

## Model

Taking inspiration from Lagrangian mechanics, where the Lagrangian is defined as $L=T-U$ with the kinetic energy *T* and potential energy *U*, we here consider a Lagrangian of the form

1 $$L=E-S,$$

where *E* denotes the *effort* and *S* the *security* in walking. It is known that the rate of metabolic energy consumption during walking on a level surface is proportional to the walking speed squared^11,24–27^. We thus assume that the effort for walking increases in proportion to the square of the walking speed *v*, and write, up to multiplicative and additive constants:

2 $$\begin{aligned} E = \left( \frac{v}{v_m}\right) ^2 , \end{aligned}$$

where $v_m$ is a constant setting the scale and making *E* dimensionless. It turns out to correspond to the maximum walking speed (see below).

On the other hand, security is related to the motivation of the pedestrian to reach a goal, while avoiding danger, and likely to depend on the walking speed and acceleration. With regard to the speed, it is reasonable to assume that the pedestrian should feel safe when she/he can cross the gap at high speeds. Measuring the speed in units of $v_m$, we thus postulate that the drive to move forward is described by the linear term $v/v_m \,(<1)$, with higher-order terms neglected. As for the acceleration, we assume a biomechanically preferred degree: The pedestrian prefers to accelerate at the preferred rate or not to accelerate at all (i.e., walking at a constant speed), and avoids accelerating in the intermediate range. Such a tendency as to the acceleration *a* is taken into account by a function *g*(*a*) (again up to multiplicative and additive constants), which we presume to be a function with zeros at $a=0$ and $a_m$ which is convex in between so that $g(a)<0$ when $0<a<a_m$. This form reflects the preference for constant speeds or high accelerations. Incorporating these two terms leads to the security in the form

3 $$\begin{aligned} S = \frac{v}{v_m} + g(a), \end{aligned}$$

We discuss two different possible choices for *g*(*a*) below. The difference between Eqs. (2) and (3) then gives the Lagrangian.

Note that we are assuming no dependence of effort or security on the position *y*. Regarding effort, this means that we are assuming a uniform and level walking terrain; Eq. (2) could be generalized to include non-uniform terrain (e.g., with some areas having gradient surfaces, which should change the energy expenditure^27^), but this is not considered here. As for security, the pedestrian would surely feel less safe in the middle of the crosswalk than elsewhere. By imposing boundary conditions, however, we are already placing a positional constraint; we thus reason that the sense of safety is fully accounted for by the speed at which the pedestrian is passing through the gap (i.e. the first term in Eq. 3). Accordingly, the Lagrangian becomes independent of the position *y* and depends only on the magnitudes of the velocity, i.e., speed ${\dot{y}}\equiv v$, and of the acceleration $\ddot{y}\equiv a$. Without loss of generality, we may assume that $v>0$ by choosing a reference frame in which the pedestrian is moving forward. Further, we suppose that the pedestrian does not decelerate until crossing, which implies $a \ge 0$. The case that the pedestrian decelerates ($a<0$) in the course of crossing is considered in Discussion.

Before we discuss the specific form of *g*(*a*) in Eq. (3), we derive a simplified form of the Euler–Lagrange equation under the given conditions. The stationary path for a Lagrangian $L=L(y, v, a)$ obeys the Euler–Lagrange equation

4 $$\begin{aligned} \frac{\partial L}{\partial y}-\frac{d}{dt} \frac{\partial L}{\partial v}+\frac{d^2}{dt^2} \frac{\partial L}{\partial a}= 0 . \end{aligned}$$

Using the fact $\partial L/\partial y =0$ and integrating with respect to time *t*, we reduce Eq. (4) to

5 $$\begin{aligned} \frac{\partial L}{\partial v}-\frac{d}{dt} \frac{\partial L}{\partial a} + c_1 =0 \end{aligned}$$

with an integration constant $c_1$. The chain rule, together with the fact $\partial L/\partial t =\partial L/\partial y =0$, yields $dL/dt=\partial L/\partial t + v \partial L/\partial y + a\partial /L\partial v + {\dot{a}}\partial L/\partial a = a\partial L/\partial v + {\dot{a}}\partial L/\partial a$ , which is substituted into Eq. (5) to obtain

6 $$\begin{aligned} \frac{dL}{dt} - {\dot{a}} \frac{\partial L}{\partial a} - a\frac{d}{dt} \frac{\partial L}{\partial a} +c_1 a = \frac{d}{dt} \left[ L - \left( a\frac{\partial L}{\partial a} \right) + c_1 v \right] = 0 \end{aligned}$$

Then Eq. (6), upon integration, obtains the form

7 $$\begin{aligned} L-a\frac{\partial L}{\partial a} + c_1 v - c_2 =0 , \end{aligned}$$

where integration constants $c_2$ and $c_1$ may be absorbed into *L* as an overall additive constant and as an multiplicative coefficient of the first term of Eq. (3). We thus set, without loss of generality, $c_1 = c_2=0$ to obtain

8 $$\begin{aligned} L-a\frac{\partial L}{\partial a} =0. \end{aligned}$$

We now consider two specific cases of *g*(*a*) that yield analytic solutions of the simplified Euler–Lagrange equation [Eq. (8)].

### Case 1: Quadratic form

**Figure 1:**
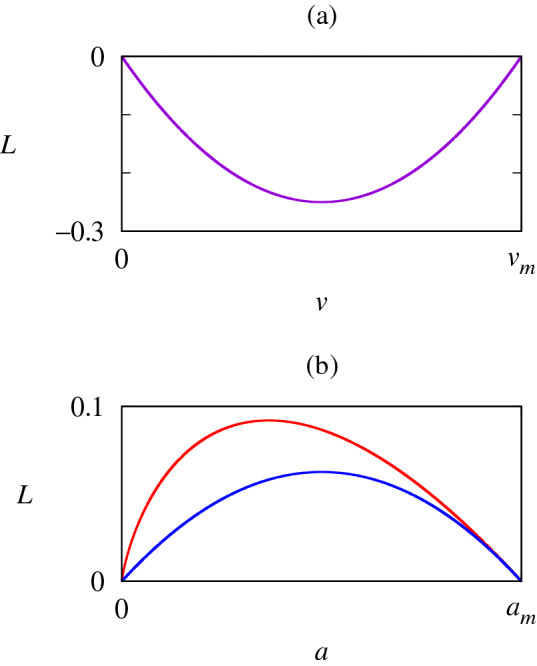
(**a**) Dependence of the Lagrangian *L* on (**a**) the speed *v* (in the absence of acceleration, $a =0$). (**b**) Dependence of *L* on *a* (for $v=0$) for the quadratic form (blue) and the logarithmic form (red).

The simplest convex function one can consider is the quadratic equation $g(a)=-(a/a_m)(1-a/a_m)$. The corresponding Lagrangian reads

9 $$\begin{aligned} L = \left( \frac{v}{v_m} \right) ^2 - \frac{v}{v_m} + \frac{a}{4a_m}\left( 1- \frac{a}{a_m} \right) , \end{aligned}$$

where the factor 1/4 on the second term, measuring the relative contributions of the speed and the acceleration, has been determined via fitting to experimental data (see Experimental Results). The dependence of *L* on *v* is illustrated in Fig. 1a whereas its dependence on *a* in the quadratic case is plotted with a blue curve in Fig. 1b. Substitution of Eq. (9) into Eq. (8) results in

10 $$\begin{aligned} \frac{dv}{dt} = 2a_m \sqrt{\frac{v}{v_m}\left( 1-\frac{v}{v_m}\right) }. \end{aligned}$$

Equation (10) has unstable fixed points at $v=0$ and $v_m$. Provided $0\le v \le v_m$, we obtain the solution

11 $$\begin{aligned} v(t) = v_m\cos ^2\left( \frac{t}{4\tau } -\frac{\pi }{4}\right) , \end{aligned}$$

which oscillates between the fixed points with $\tau \equiv v_m/4a_m$ measuring the duration of each acceleration and deceleration interval. Under appropriate boundary conditions, we can reason that the least action path begins at rest ($v=0$), accelerates to $v_m$ following Eq. (11) during a time interval centered at time $t=t_a$, and then continues to move with constant velocity $v=v_m$. This is described by the following piecewise function:

12 $$\begin{aligned} v(t) = {\left\{ \begin{array}{ll} 0, &{} t-t_a\le -\pi \tau \\ v_m\cos ^2((t-t_a)/4\tau - \pi /4) , &{} -\pi \tau< t-t_a < \pi \tau \\ v_m, &{} t-t_a\ge \pi \tau , \end{array}\right. } \end{aligned}$$

which, upon integration, leads to

13 $$\begin{aligned} y(t) = {\left\{ \begin{array}{ll} y_0, &{} t-t_a\le -\pi \tau \\ y_0 + \frac{\tau v_m}{2} \left( \pi + \frac{t-t_a}{\tau } -2\cos \left[ \frac{t-t_a}{2\tau }\right] \right) , &{} -\pi \tau< t-t_a < \pi \tau \\ y_0 + v_m (t-t_a), &{} t-t_a\ge \pi \tau . \end{array}\right. } \end{aligned}$$

Equations (12) and (13) obey the Euler–Lagrange equation at all points, but they exhibit singularities in the higher derivatives at times $t=t_a \pm \pi \tau$.

### Case 2: Logarithmic form

As a second case, we take $g(a)=(a/a_m)\log (a/a_m)$, which yields

14 $$\begin{aligned} L = \left( \frac{v}{v_m} \right) ^2 - \frac{v}{v_m} - \frac{a}{4a_m}\log \left( \frac{a}{a_m} \right) , \end{aligned}$$

again with a factor of 1/4. The dependence of *L* on *a* in the logarithmic case is plotted with a red curve in Fig. 1b. Unlike the quadratic equation, this form of *g*(*a*) is asymmetrical and steeper on the left side. Plugging into Eq. (8) results in the logistic equation

15 $$\begin{aligned} \frac{dv}{dt} = \frac{4a_m v}{v_m}\left( 1-\frac{v}{v_m}\right) , \end{aligned}$$

which carries the solution for the speed:

16 $$\begin{aligned} v(t) = \frac{v_{m}}{2} \left[ \tanh \left( \frac{t-t_a}{2\tau } \right) +1 \right] , \end{aligned}$$

where $\tau$ is defined the same way as above, again measuring the duration of acceleration, and the integration constant $t_a$ determines the timing of acceleration. Note that $v_m$ in Eq. (16) indeed corresponds to the maximum walking speed. Further, differentiation of Eq. (16) with respect to *t* manifests that the maximum acceleration is given by $a_m$. Integrating Eq. (16), we also obtain the position as a function of time:

17 $$\begin{aligned} y(t) =y_0+ v_{m}\tau \log \left[ 1+ \exp \left( \frac{t-t_a}{\tau }\right) \right] . \end{aligned}$$

Unlike Eq. (13), Eq. (17) is free of singularities. Instead, however, the speed reaches neither exactly 0 nor $v_m$, and has thus the disadvantage of being an approximation (albeit the error decays exponentially).

## Experimental results

### Data collection

To verify the validity of each case of the model, we make a comparison with the data obtained from a virtual reality road-crossing experiment. In this experiment, human participants walked on a customized treadmill (of dimensions 0.67 m wide, 1.26 m long, and 1.10 m high) with four magnetic counters that track movements. A Velcro belt connected to the treadmill was worn for suppression of vertical and lateral movements, and a handrail was placed for safety. Each participant wore a commercial virtual reality headset connected to a standard desktop PC. The headset portrayed a realistic view of a typical crosswalk in Korea in $1280\times 800$ resolution stereoscopic visual images which shift in real time according to the participant’s steps and head turns. Participants from the two age groups were recruited as follows: children were recruited from an elementary school in a middle-class district of Gunsan City, Republic of Korea, while young adults were recruited through a Kunsan National University social media post. Participants were required to have normal or corrected-to-normal vision, no persistent problems with dizziness, and no history of serious traffic accidents. If a participant experienced motion sickness, the experiment was immediately halted and the participant excluded from the data. In this way, two adults were excluded from the experiment. Sixteen children (of age $12.2 \pm 0.8$ yrs, i.e., mean age 12.2 years and standard deviation 0.8 years) and sixteen adults (of age $22.8 \pm 2.6$ yrs) participated in the experiment and were included in the data set. Informed written consent was obtained from every individual participant; for each juvenile participant, informed written consent was obtained from the parent or legal guardian. The experiment was conducted according to the Declaration of Helsinki, and the protocol was approved by the Ethics Committee of the Kunsan National University Research Board. Details of the experiment can be found in Chung *et al*^22,23^.

**Figure 2:**
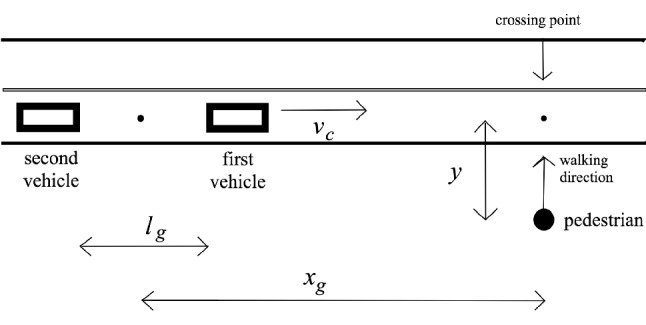
Schematic diagram of the crossing environment. The two boxes on the road depict two vehicles facing right, which move forward at constant speed $v_c$. The circle depicts the pedestrian, who walks in the perpendicular direction (shown by the arrow). The gap between the two cars is $l_g$ in length while the distance between the pedestrian and the midpoint of the gap is $x_g$. The position of the pedestrian is measured by the distance *y* from the crossing point.

Figure 2 presents a schematic diagram of the crossing simulations viewed from above. While the two parallel vehicles are moving at equal constant speed $v_c=30$ km/h, the pedestrian attempts to cross the road in the perpendicular direction. The paths of the pedestrian and of vehicles intersect at the crossing point. The pedestrian is instructed to cross between the two vehicles if possible. The empty space between the two vehicles, called the gap, is set to be $l_g=25$ m in length. The distance between the midpoint of the gap and the intersection point, denoted by $x_g$, has the initial value 33.3 m, so that the gap center reaches the crossing point in 4 s. The position of the pedestrian is measured by the distance *y* from the crossing point taken as the origin and is recorded to generate positional time series. The initial position $y_0$ is set to be $-4.5$ m.

### Fitting results

**Figure 3:**
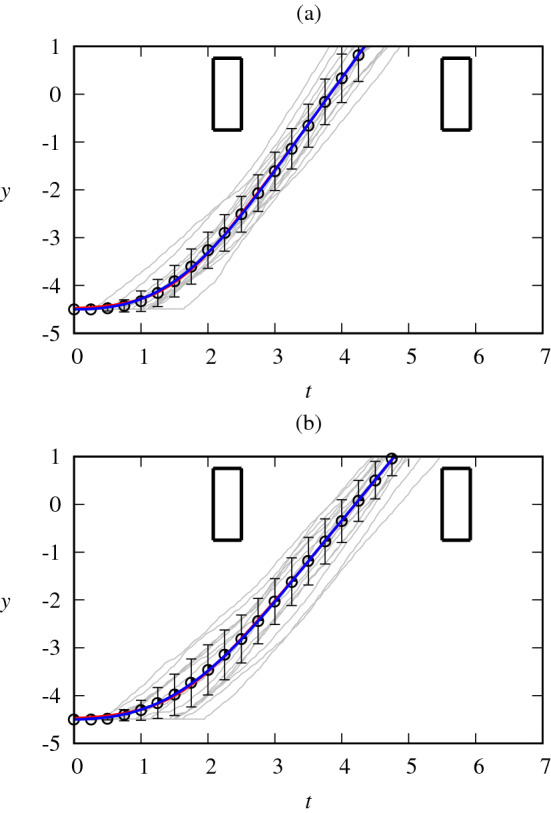
Fitting results together with data for (**a**) adults and (**b**) children, displaying the position *y* (in meters) versus time *t* (in seconds). Circles and error bars indicate averages and standard deviations of positions, respectively. Blue and red lines correspond to Eqs. (13) and (17) fitted to the averaged data, respectively. The blue and red lines overlap significantly. Individual time series data are plotted in grey and rectangles represent vehicles.

We fit Eqs. (13) and (17) to the data, making use of $v_m$, $\tau$, and $t_a$ as fitting parameters. When Eq. (13) was fit to the data, the root-mean-square deviation (RMSD) turned out to be 0.052 m on average, with the standard deviation 0.022 m and the maximum RMSD of 0.10 m. Meanwhile, when Eq. (17) was fit to the data, the RMSD of 0.056 m was obtained on average, with the standard deviation 0.024 m and the maximum RMSD of 0.12 m. In all fits, the coefficient of determination was found to be close to unity: $0.996 \le R^2 \le 0.999$. It was thus concluded that either model function makes a description of each individual crossing with high accuracy, and no significant difference between the two was observed. All-time series are plotted in Fig. 3, which manifests that overall, data (thin gray lines) fit closely to the model.

There were individual variations in the slope $v_m$ and the acceleration timing $t_a$, resulting in a spread of the data as seen in Fig. 3. Taking the average of the position data in 0.25 s increments, we obtain the average behavior of each collective group and plot the averages and standard deviations also in Fig. 3. The thick red and blue lines depict Eqs. (13) and (17) fitted to the averaged position data, with the fitting parameters given in Table 1. Note first that the averaged data also display a good fit for both cases of the model. By plotting the two age groups separately, we observe a difference in the slope. Accordingly, $v_m$ takes different fitting values: The adult group has a higher value by 0.24 m/s (see Table 1). Other parameters do not differ significantly.

**Table 1:** Fitting parameters $v_m$, $t_a$, and $\tau$ for the group-averaged data, together with Lagrangian constant $a_m$, in each model.

Model	Age group	$v_m$ (m/s)	$\tau$ (s)	$t_a$ (s)	$a_m$ (m/$\hbox {s}^2$)
Quadratic	Adults	1.94	0.54	1.50	0.90
	Children	1.70	0.59	1.55	0.72
Logarithmic	Adults	1.94	0.43	1.51	0.32
	Children	1.70	0.47	1.57	0.27

## Discussion

We have presented a model for goal-directed human walking behavior, based on a principle of least action. The approach can be considered as a generalization of the principle of least effort, incorporating another term called security. Walking behavior results from the assumption of three simple terms making up effort and security. The resulting equations have been found to fit experimental data from a virtual reality road-crossing experiments. While the equations were derived from a Lagrangian, an equivalent Hamiltonian formulation can also be constructed (see Supplementary Information).

It is not conceivable that our simple model captures the full complexity of the complex biomechanics and psychology involved in walking. The model treats the walker as a self-propelling particle and thus provides rather a coarse approach compared with biomechanical studies^28^. However, we presume that the form of Eq. (1) should contain all the essential features of goal-directed walking, including psychological factors. For example, the pendulum-like mechanics of walking is implicit in the form of energy consumption given by Eq. (2). This approach may thus be useful in the study of pedestrian trajectories.

For validation of the model, an experimental setup was employed which imposed a gap interception task on the participant. The results show that the participant chooses the path of least action as defined by the model. However, while the initial conditions are constrained, the experiments force the participants not into a single point but into a spatiotemporal range (the gap). Accordingly, there are individual variations in the endpoint the participant chooses. In addition, we expect that physiological differences lead to differences in constants $v_m$ and $a_m$, which could result in variations in the least-action path even with the same boundaries. This is made apparent in the difference in $v_m$ between the age groups, which is manifested by different values of $v_m$ and $a_m$ in the Lagrangian. A detailed examination of the effects of various other crossing conditions (e.g. the initial position of the pedestrian, the gap length, and the vehicle speed) on fitting parameters can be found in Kim *et al.*^29^.

One may note the limitations of using a treadmill, which may change walking behavior and also constrains the participant to walk in a straight line. Additional walking simulations have been done in which the participants walk freely in a room, with sensors used to detect their positions. The results were again consistent with the model (data not shown). However, an additional dimension is added due to the freedom in the walking direction. There were no interesting features in the dynamics of the angle, which was generally held constant. The choice of the angle exhibits also individual variations; this is beyond the scope of the current model.

Note also that the model has been limited to the case of positive acceleration ($a>0$). The quadratic Lagrangian in Eq. (9) can already describe negative accelerations, as a piecewise function can be constructed with a deceleration event from velocity $v_m$ to 0 due to the oscillatory form of Eq. (11). For the logarithmic Lagrangian in Eq. (14), with negative accelerations ($a<0$) allowed, we may assume that the security feeling of the pedestrian should depend only on the magnitude (regardless of the sign), and put the absolute value |*a*| in place of *a* in Eq. (3). This reverses the solution over time: The speed begins at $v_m$, then decreases to zero. Such a time-reversed solution describes the deceleration event after the pedestrian has reached a destination. Under some experimental conditions (not shown here), the participant walked forward, stopped, and then accelerated again to cross the gap^29^. The stopping behavior between walking can be seen as a deceleration event described by the model with $a<0$. For simplicity, this analysis is left out of the present study.

We note that our model has similarities with other models of pedestrian behavior, e.g., in Guy *et al.*^7^, which also defines effort as the metabolic energy consumption. Our model differs from those previous models in that it includes additional terms (security) affecting the trajectory and also in that it produces an analytical solution for the entire walking trajectory, which is possible due to the simplicity of the walking task. Guy *et al.* instead simulate collision avoidance in crowds by restricting the direction of movement based on the environment at each simulation step. The least action model may be extended to include interactions with other pedestrians and walking directions; this is left for future study.

## Supplementary Information

Supplementary Information.
