# Supplementary material for: A least action principle for interceptive walking
Source: Sci Rep. 2021 Jan 26;11:2198. doi: 10.1038/s41598-021-81722-6 (PMC7838306; doi:10.1038/s41598-021-81722-6)
Supplement: Supplementary file 1 — Supplementary Information. [file 41598_2021_81722_MOESM1_ESM.pdf]

A least action principle for interceptive walking

Soon Ho Kim[1,2], Jong Won Kim[3], Hyun Chae Chung[4], and MooYoung Choi[1,*]

[1]Department of Physics and Astronomy and Center for Theoretical Physics, Seoul National University, Seoul 08826, Korea
[2]Present address: Brain Science Institute, Korea Institute of Science and Technology, Seoul 02792, Korea
[3]Department of Healthcare Information Technology, Inje University, Gimhae 50834, Korea
[4]Department of Sports Science, Kunsan National University, Gunsan 54150, Korea
[*]mychoi@snu.ac.kr

Supplementary Note: Hamiltonian formulation

The Lagrangian formulation immediately suggests that a Hamiltonian may also be constructed. The proposed Lagrangian differs from those typical in classical mechanics in that it contains a derivative of order higher than one. Lagrangians of such form have been studied, and instabilities that arise from unavoidable linear terms in the corresponding Hamiltonian functions have been cited as reasons that nature is not described by higher-order Lagrangians in fundamental theory.[1,2] We appeal to the phenomenological nature of the model and disregard these instabilities here. Making use of Ostrogradsky's construction for third-order Lagrangians[3]

$$\begin{aligned} Q_1 &\equiv x \\ Q_2 &\equiv \dot{x} \\ P_1 &\equiv \frac{\partial L}{\partial \dot{x}} - \frac{d}{dt}\frac{\partial L}{\partial \ddot{x}} \\ P_2 &\equiv \frac{\partial L}{\partial \ddot{x}} \end{aligned} \tag{A1}$$

and performing the Legendre transform, we construct the Hamiltonian as follows:

$$H(Q_1, Q_2, P_1, P_2) = P_1 Q_2 + P_2 A(Q_1, Q_2, P_2) - L(Q_1, Q_2, A), \tag{A2}$$

where A is the acceleration function expressed in terms of the canonical coordinates.

In the quadratic case, this results in a Hamiltonian of the form

$$H = \frac{Q_2}{v_m} - \frac{Q_2^2}{v_m^2} + Q_2 P_1 - \frac{a_m}{2} P_2 - a_m^2 P_2^2 + \frac{3}{16}, \tag{A3}$$

while in the logarithmic case, we have

$$H = \frac{Q_2^2}{v_m^2} - \frac{1}{4} e^{-4a_m P_2 - 1} + Q_2 P_1 - \frac{2Q_2^2}{v_m^2} - \frac{1}{v_m}. \tag{A5}$$

The Hamiltonians obtained in this way provide indeed conserved quantities.

References

1. Woodard, R. P. Avoiding dark energy with 1/R modifications of gravity. *Lect. Notes Phys.* **720**, 403 (2007).
2. Motohashi, H. & Suyama, T. Third order equations of motion and the Ostrogradsky instability. *Phys. Rev. D* **91**, 085009 (2015).
3. Ostrogradsky, M. Mémoires sur les équations différentielles, relatives au probleme des isopérimetres. *Mem. Ac. St. Petersbourg* **VI**, 385 (1850).
